# Protein hydrolysates in sports nutrition

**DOI:** 10.1186/1743-7075-6-38

**Published:** 2009-09-28

**Authors:** Anssi H Manninen

**Affiliations:** 1 Manninen Nutraceuticals Oy, Hajottamotie 11, 90550 Oulu, Finland

## Abstract

It has been suggested that protein hydrolysates providing mainly di- and tripeptides are superior to intact (whole) proteins and free amino acids in terms of skeletal muscle protein anabolism. This review provides a critical examination of protein hydrolysate studies conducted in healthy humans with special reference to sports nutrition. The effects of protein hydrolysate ingestion on blood amino acid levels, muscle protein anabolism, body composition, exercise performance and muscle glycogen resynthesis are discussed.

## Introduction

Proteins and amino acids are an important part of a diet, and as such, have been the subject of a great deal of discussion and controversy, especially among strength/power athletes. More than 15 y ago some bodybuilding magazines suggested that protein hydrolysates providing mainly di- and tripeptides are superior to intact (whole) proteins and free amino acids in terms of skeletal muscle protein anabolism.

This proposition was apparently based on early studies suggesting that protein hydrolysates were more effectivelty utilized in rats than intact proteins or free amino acids (e.g. [1]). Obviously, rats are not small versions of humans, so sports nutritionists remained highly skeptical. Nevertheless, some major sports supplement manufacturers included protein hydrolysates in many of their products. More recently, protein hydrolysate-containing products specifically formulated for post-exercise recovery have gained some popularity [2].

This review provides a critical examination of the protein hydrolysate studies conducted in healthy humans with special reference to sports nutrition. Some animal studies are also discussed.

## Basic facts about protein hydrolysates

Protein hydrolysates are produced from purified protein sources by heating with acid or, preferably, addition of proteolytic enzymes, followed by purification procedures. Each protein hydrolysate is a complex mixture of peptides of different chain length together with free amino acids, which can be defined by a global value known as degree of hydrolysis (DH), which is the fraction of peptide bonds that have been cleaved in the starter protein [3]. However, even the exact information on DH cannot not tell us the whole story, as two protein hydrolysates made by different methods (e.g., oligopeptides/significant free amino acids *vs*. mainly dipeptides and tripeptides) may have a similar degree of hydrolysis even though their absorption kinetics are likely quite different. Consequently, all protein hydrolysates are certainly not created equal.

## Absorption of protein hydrolysates

It is generally accepted that only di- and and tripeptides, which remain after luminal and brush-border peptidase digestion, are absorbed intact [4]. Tetrapeptides and higher peptides appear to require prior brush-border hydrolysis before their hydrolysis products can be absorbed [4]. Early studies by Grimble and colleagues demonstrated that whey, egg and casein protein hydrolysates containing mostly di- and tripeptides are more rapidly absorbed than those based on longer peptides [5-7]. In their studies, the contents of di- and tripeptides were carefully analyzed. These results led Grimble to conclude that the proportion of di- and tripeptides determines absorption kinetics of protein hydrolysates [4]. In addition, Adibi and Morse established that *1) *the disappearance of tetraglycine in the human jejunum is accomplished principally by hydrolysis by brush border oligopeptidases; *2) *the rate limiting step in the uptake of glycine from tetraglycine or longer peptides is due to hydrolysis of these peptides to absorbable products (i.e., di- and triglycine); and *3) *the rate of glycine uptake is markedly greater from di- and triglycine than from free glycine [8].

Therefore, it is generally accepted that protein hydrolysates containing mostly di- and tripeptides are absorbed faster than intact proteins. However, some researchers have recently reported seemingly conflicting results. A study by Farnfield *et al*. examined the plasma amino acid responses to intact whey protein isolate and hydrolyzed whey protein isolate [9]. After an overnight fast, their subjects consumed a 500 ml beverage containing 25 g of protein, and blood was taken at rest and then every 15 min for 2 h post ingestion. The authors reported, quite suprisingly, that intact whey protein results in a rapid absorption of amino acids into the blood compared with the hydrolyzed whey protein. Unfortunately, the authors did not provide any information on whey protein hydrolysate used in this study, other than that it was produced by Dairy Farmers, a company which does not appear to be a large-scale producer of protein hydrolysates.

A more recent study by Power *et al*. used aextensively hydrolyzed whey protein produced by a major whey protein hydrolysate manufacturer [10]. Sixteen healthy men ingested a 500 ml solution containing either 45 g of intact whey protein or whey protein hydrolysate. When analyzed over the 3 h period, the estimated rate of gastric empting was not significantly altered by industrial hydrolysis of the protein, In addition, the rate of apperance of branched chain amino acids was not significantly altered by hydrolysis of whey protein. This may be explained by the fact that protein drinks were ingested on empty stomach after an overnight fast. Alternatively, whey protein is rapidly absorbed protein even in its intact form, so perhaps hydrolysis simply does not significantly affect its absorption kinetics.

Nevertheless, maximum plasma insulin concentration was 28% greater following ingestion of the whey protein hydrolysate compared to the intact whey protein, leading to a 43% increase in the 3 h area under curve of insulin for the whey protein hydrolysate. Thus, the stronger insulinotopic effect of whey protein hydrolysate appears to be unrelated to gastric emptying of the protein solution. Calbet and Holst reported that whey and casein protein hydrolysates elicited ~50% more gastric secretion than respective intact proteins, which was accompanied by higher plasma concentrations of glucose-dependent insulinotropic polypeptide (GIP) during the first 20 min of the gastric emptying process [11]. As its name implies, GIP facilitates insulin-release from pancreatic beta-cells. Interestingly, there is evidence to suggest that GIP is a growth and antiapoptotic factor for pancreatic beta-cells [12]. In type 2 diabetes, GIP no longer modulates glucose-dependent insulin secretion, even at supraphysiological plasma levels, and therefore GIP incompetence is detrimental to pancreatic beta-cell function, especially after eating [13]. Whether increased GIP secretion induced by protein hydrolysate ingestion offers any benefits for healthy athletes remains to be determined.

Certainly, hydrolysis can facilitate absoption of casein protein (the main component of whole milk protein), which is a slowly absorbed protein in its intact form. In a recent study by Koopman *et al*., subjects received a 350 ml bevarage containing 35 g of either intact casein protein or extensively hydrolyzed casein protein [14]. The results showed that casein hydrolysate ingestion induced ~25-50% higher plasma amino acid peaks than intact casein ingestion.

It is important to realize that the studies by Farnfield *et al*. and Power *et al*. measured plasma amino acid response, which is not a good variable, as it is not indicative for amino acid flux, and thus, not for amino acid absorption. The study by Koopman *et al*. used labeled proteins; therefore, in their case, the plasma response is predictive for amino acid absorption.

## Utilization of protein hydrolysates

A study by Moriarty *et al*. is frequently cited, concluding that the molecular form of protein elicits no difference in nitrogen balance in healthy humans [15]. This was hardly surprising as the basis of question relates to the *rapidity *of absorption of amino acids. In the Moriarty study this was not an issue because feeding patterns were essentially unconstrained. Under conditions of rapid intestinal infusion, amino acids from protein hydrolysates appear in the portal circulation faster than even free amino acids [16].

The most sophisticated study to date demonstrated that a 35 g dose of rapidly absorbed casein hydrolysate is ~30% more effective in stimulating skeletal muscle protein synthesis than intact casein when measured over the 6 h period [13]. Based on plasma amino acid and insulin peaks, it can be speculated that the difference would have been larger if the study period would have been 2 or 3 h. Rapid absorption of amino acids appears to decrease splanchnic extraction, which likely explains the greater systemic amino acid levels and therefore greater anabolic effects. This notion is supported by a study by Paddon-Jones *et al*., who reported that a oral supplement containing 30 g of carbohydrate and 15 g of essential amino acids induces a substantially greater anabolic effect than ingestion of a mixed meal containing a similar amount of essential amino acids [17]. Also, Tang *et al*. showed that ingestion of whey protein hydrolysate results in a larger increase in blood amino acids and mixed muscle protein synthesis than soy protein or casein both at rest and after resistance exercise [18].

Collectively, these studies strongly suggest that ingestion of a fast-acting protein hydrolysate and/or amino acid supplement results a less efficient uptake by the splanchnic bed and therefore increases the magnitude of the acute increase in amino acids in the systemic circulation that are available for muscle protein anabolism. The splanchnic bed comprises *1) *the liver and *2) *the portal-drained viscera (PDV), which include the stomach, intestines, pancreas, and spleen. First-pass extraction by splanchnic tissues describes the proportion of ingested amino acids that is sequestered during its initial transit through the splanchnic bed and thus not appearing in systemic blood [19]. Although largely ignored by the authors of sports nutrition textbooks, the extraction of amino acids by the intestine have a critical influence on their availability to peripheral tissues and therefore, on whole body protein metabolism. In fact, the PDV account for 20 to 35% of whole-body protein turnover and energy expenditure [19]. Thus, the notion than an amino acid is an amino acid no matter how administered is clearly fallacious. The kinetics of absorption of amino acids can substantially modulate their ability to stimulate muscle protein anabolism.

## Protein hydrolysates, body composition and muscular performance

Cribb *et al*. investigated the effects of supplementation with two protein supplements on muscle strength and body composition during a 10 wk, supervised resistance training program [20]. In a double-blind protocol, recreational male bodybuilders supplemented their normal diet with either hydrolyzed whey protein isolate or casein (1.5 g/kg body wt/d) for the duration of the program. The authors reported that the whey protein hydrolysate group achieved substantially greater gains in muscle strength and lean body mass (LBM) compared to the casein group. It should be noted, however, that the whey protein hydrolysate group started at a higher weight with a narrower variance, which may have exaggerated differences in LBM gains.

Buckley *et al*. examined whether hydrolyzed whey protein isolate speeds recovery more effectively than intact whey protein isolate following eccentric exercise [21]. The subjects performed 100 maximal eccentric contractions of their knee extensors and then consumed either 25 g of hydrolyzed whey protein isolate or intact whey protein isolate. Interestingly, peak isometric torque was recovered fully in 6 h in the whey protein hydrolysate group, while it remained suppressed in the intact whey protein group.

In summary, whey protein hydrolysate appears to offer some ergogenic benefits, but more research is clearly needed before firm conclusions can be drawn.

## Intra-exercise protein hydrolysate ingestion

Although carbohydrates and lipids supply most of the energy needs during exercise, human skeletal muscle can also oxidize at least seven amino acids, namely leucine, isoleucine, valine, glutamate, asparagine, aspartate and alanine, providing additional free energy to fuel muscle contraction. In addition, amino acid catabolism during exercise increases citric acid cycle intermediates and therefore supports carbohydrate and lipid catabolism.

Rapidly absorbed protein hydrolysates may be expecially suitable for intra-exercise consumption. A well designed study by Beelen *et al*. examined the effect of protein hydrolysate co-ingestion with carbohydrate on muscle protein anabolism during resistance-type exercise [22]. Importantly, the subjects in their study were investigated in a postprandial state, reflecting a real-life situation. The subjects received a bolus of test drink before and every 15 min during exercise, providing 0.15 g/kg/h high-glycemic carbohydrates with or without 0.15 g/kg/h casein hydrolysate. The results indicated that mixed muscle protein fractional synthetic rate was substantially higher after protein hydrolysate co-ingestion. Similar findings were reported during a combined endurance and resistance exercise session [23].

Saunders *et al*. investigated whether a carbohydrate plus casein hydrolysate beverage improves time-trial performance *vs*. a traditional carbohydrate beverage delivering ~60 g of carbohydrates per h [24]. Male cyclists completed two computer-simulated 60-km time trials consisting of 3 laps of a 20-km course concluding with a 5-km climb (~5% grade). In a double-blind fashion, the subjects consumed 200 ml of carbohydrate (6%) or carbohydrate plus casein hydrolysate (6% plus 1.8% protein hydrolysate) every 5 km and 500 ml of beverage immediately after exercise. The conclusion was that late-exercise time-trial performance was enhanced with carbohydrate plus casein hydrolysate beverage ingestion compared with a traditional beverage containing only carbohydrate. In addition, carbohydrate plus casein hydrolysate ingestion prevented increases in plasma creatine kinase and muscle soreness after exercise.

In summary, intra-exercise protein hydrolysate ingestion seems to offer substantial benefits. However, it is currently not know whether protein hydrolysate ingestion offers advantages over intact protein ingestion.

## Protein hydrolysates and muscle glycogen resynthesis

Employing rat L6 myotubes and isolated epitrochlearis muscles, Morifuji *et al*. showed that branched chain amino acid-containing bioactive dipeptides in whey protein hydrolysate (Ile-Val, Leu-Val, Val-Leu, Ile-Ile, Leu-Ile, Ile-Leu) significantly stimulate glucose uptake in the L6 myotubes, while Ile-Leu, the main component in whey protein hydrolysate, stimulates glucose uptake also in isolated muscles [25]. Another study by Morifuji *et al*. compared the effects of different proteins on post-exercise glycogen resynthesis [26]. Immediately after the glycogen-depleting exercise, male Sprague-Dawley rats were given either glucose alone, glucose plus whey protein, glucose plus whey protein hydrolysate, glucose plus casein hydrolysate or glucose plus branched-chain amino acid (BCAA). The results revealed that whey protein hydrolysate ingestion induced significant increases in skeletal muscle glycogen levels compared with other protein sources or BCAA.

Thus, whey protein hydrolysate appears to enhance the effects of carbohydrate ingestion on post-exercise muscle glycogen resynthesis. Whether whey protein hydrolysate stimulates muscle glucose uptake and glycogen resynthesis in athletes remains to be determined.

## Insulin secretion and skeletal muscle anabolism

The physiologically most important insulin secratogue is certainly glucose; however, the secretion of insulin can be induced by number of compounds, including amino acids. Leucine has a quite potent insulinotropic effect, but a recent study suggests that a rise in glucose concentration is necessary for leucine to stimulate significant insulin secretion [27].

Protein hydrolysate ingestion induces substantially greater insulinotropic effect than intact proteins [2], and it has been suggested that the greater insulin response contributes to muscle protein anabolism, especially after resistance exercise [2]. However, a growing body of evidence suggest that insulin is mainly permissive for muscle protein anabolism [28]. For example, Greenhaff et al. demonstrated that hyperaminoacidemia does not require hyperinsulinemia to exert a power anabolic effect in human skeletal muscle [29]. Specifically, they showed that increasing availability of amino acids and of insulin over the range of 5-167 mU/l doubled leg protein synthesis and halved leg protein breakdown without any dose-response relationship with insulin. In other words, hyperaminoacidemia has powerful anabolic effects even in face of basal insulin (~5 mU/l). Also, their results indicate that increasing insulin to 30 mU/l halved muscle protein proteolysis without further inhibition at higher doses.

More practical evidence was provided by Koopman *et al*., who showed that co-ingestion of high-glycemic carbohydrates during recovery does not further stimulate post-exercise muscle protein anabolism when a relatively large amount of casein protein hydrolysate is ingested eventhough plasma insulin responses were markedly enhanced by carbohydrates [30]. Also, Harber *et al*., reported that a high-protein/very-low-carbohydrate diet increases skeletal muscle protein anabolism despite a dramatic reduction in insulin levels [31].

However, the insulin-induced increase in muscle protein anabolism is blunted in older adults. For example, Fujita *et al*. measured leg muscle protein synthesis and amino acid kinetics in healthy, glucose-tolerant older volunteers at baseline, and during an insulin infusion at postprandial (0.15 mU/min/100 ml) or supraphysiologically high (0.30 mU/min/100 ml) doses [32]. The results revealed that muscle protein synthesis increased only in those receiving supraphysiologically high doses of insulin. Net amino acid balance across the leg improved in both groups, but a net anabolic effect was observed only with supraphysiologically high doses.

In summary, it now appears that insulin is mainly permissive rather than stimulatory for muscle protein anabolism in young adults. However, supraphysiological hyperinsulinemia appears to be necessary to stimulate muscle protein anabolism in older individuals.

## Future directions

As far as sports nutrition is concerned, protein hydrolysates, usually produced from whey or casein protein, are generally not used as meal replacements. Rather, athletes use these products to induce rapid increases in plasma amino acids around workouts (i.e., before, during and after workouts), which may maximize muscle protein anabolism and facilitate recovery. However, there are no studies comparing the effects of whey or casein protein hydrolysates and respective intact proteins on skeletal muscle anabolism in healthy athletes.

Ideally, such studies should reflect a real-life situation. That is, protein supplements should be ingested before, during and/or after exercise. Also, a realistic "strength-power diet" (i.e., a diet high in calories and protein) should be followed by the subjects. A high-quality protein hydrolysate containing mostly di-and tripeptides should be used. The proportion of di- and tripeptides appears to determine absorption kinetics, and in turn, it is the kinetics of the absorption (rather than the net absorption of amino acids) that determines the greater nutritional value of the protein hydrolysates.

## Abbreviations

BCAA: branched-chain amino acids; DH: degree of hydrolysis; GIP: glucose-dependent insulinotropic polypeptide; LBM: lean body mass; Leu: L-leucine; Ile: L-isoleucine; Val: L-valine; PDV: portal-drained viscera.

## Competing interests

The author declares that he has no competing interests.
